# Enhancing the Wisdom of the Crowd With Cognitive-Process Diversity: The Benefits of Aggregating Intuitive and Analytical Judgments

**DOI:** 10.1177/0956797620941840

**Published:** 2020-09-22

**Authors:** Steffen Keck, Wenjie Tang

**Affiliations:** 1 Department of Business Administration, University of Vienna; 2 Institute of Operations Research and Analytics, National University of Singapore

**Keywords:** decision making, judgment, cognitive processes, wisdom of the crowd, judgment aggregation, group judgments, dual-process theory, open data

## Abstract

Drawing on dual-process theory, we suggest that the benefits that arise from combining several quantitative individual judgments will be heightened when these judgments are based on different cognitive processes. We tested this hypothesis in three experimental studies in which participants provided estimates for the dates of different historical events (Study 1, *N* = 152), made probabilistic forecasts for the outcomes of soccer games (Study 2, *N* = 98), and estimated the weight of individuals on the basis of a photograph (Study 3, *N* = 3,695). For each of these tasks, participants were prompted to make judgments relying on an analytical process, on their intuition, or (in a control condition) on no specific instructions. Across all three studies, our results show that an aggregation of intuitive and analytical judgments provides more accurate estimates than any other aggregation procedure and that this advantage increases with the number of aggregated judgments.

As first noted by Galton (1907), a statistical combination of quantitative judgments made by multiple judges is typically more accurate than the judgment of a randomly selected individual judge. Importantly, the benefits of forming a crowd by statistically aggregating a number of individual judgments depend strongly on the level of independence among the errors of these judgments (e.g., Lamberson & Page, 2012; Larrick & Soll, 2006; Mannes, Soll, & Larrick, 2014; Simmons, Nelson, Galak, & Frederick, 2011). Specifically, if judges’ errors are mostly independent from each other, their judgments will frequently bracket the correct answer, and errors will cancel out. In contrast, when errors are systematically correlated—that is, when judges collectively either under- or overestimate the true value—bracketing will occur only rarely, and thus judgment aggregation loses most of its benefits. To address this problem of correlated errors, we propose heightening the level of independence between individual judgments by manipulating the cognitive process that individuals use to form their judgments. In particular, building on dual-process theory (e.g., Kahneman, 2011; Sloman, 1996), we argue that forming a crowd with a high level of cognitive-process diversity (by combining judgments based on an intuitive cognitive process and those based on an analytical cognitive process) will be more beneficial than combining judgments based on the same cognitive process.

## The Benefits of Cognitive-Process Diversity

Dual-process theory posits that there are two distinct cognitive processes that individuals rely on to form judgments: An *intuitive* process, typically described as preconscious, fast, and operating in a holistic manner, and an *analytical* process, characterized as slow, deliberative, rule-governed, and conscious. Importantly, the literature also suggests that there is no universal advantage of one cognitive process over another (e.g., Plessner & Czenna, 2008). Instead, because they draw on different sources of information and decision rules to form a judgment, each process is likely to have distinct advantages and disadvantages. Specifically, whereas intuitive thinking typically relies on only partial information that spontaneously comes to mind when processing a stimulus, analytical thinking tends to involve consideration of different aspects of a particular problem and a deliberate cognitive search for additional information (e.g., Dane & Pratt, 2007; Hogarth, 2010). Moreover, the information that intuitive thinking relies on is usually learned implicitly (e.g., from direct experience), whereas analytical thinking relies more strongly on explicitly learned knowledge (Hogarth, 2010). Finally, the two systems also differ with respect to the mechanism by which information is used to form judgments and decisions: In an intuitive mode of thinking, individuals tend to focus on a single holistic cue that encompasses all information at hand to form a judgment, whereas in an analytical mode, thinking is typically based on an explicit aggregation of several unitary cues, and different cues are weighted on the basis of their perceived validity (e.g., Dane, Rockmann, & Pratt, 2012; Epstein, 2010; Hogarth, 2010). Hoffmann, von Helversen, and Rieskamp (2013) found results consistent with this argument: Generally, when individuals are placed under cognitive load and thus are potentially more prone to rely on an intuitive rather than an analytical thinking process, their judgments are more likely to be based on the average value of similar, previously encountered items rather than on a process that combines cues using linear rules.

**Statement of Relevance**

Psychological scientists have long known that the wisdom of many people aggregated together (a crowd) is often better than the wisdom of any one individual. Yet the judgments made by crowds of people are not perfect and can even be highly inaccurate. In this research, we tested a means of improving crowd wisdom by diversifying the bases on which the individuals that made up the crowd formed their judgments—either through intuition or through analytical thinking. We found that crowds with a high level of diversity in the cognitive processes they used made better judgments than crowds with lower levels of diversity. We also found that the magnitude of the benefits increased with crowd size. This work suggests that we can improve crowd wisdom not by selecting individuals who each make better judgments but by aggregating the judgments of people who “go with their gut” and of people who carefully think through the problem.

There have been a number of critiques concerning the conceptual clarity and potential lack of predictive power of dual-process theory (e.g., Keren & Schul, 2009). Of particular relevance to our main argument is Kruglanski and Gigerenzer’s (2011) contention that analytical judgments might frequently be based on the same (simple) rules as intuitive judgments if such rules are judged to be of high validity. Importantly, even though differences between intuitive and analytical judgments might thus not always be as clear as what dual-process theory would predict, this line of reasoning would still suggest that, at least on average, analytical judgments will be more likely to be based on complex (vs. simple) rules than intuitive judgments are.

In summary, we propose that because analytical and intuitive processes at least partially rely on different information and mechanisms to form judgments, they can be expected to produce errors that are less systematically correlated with each other—compared with judgments that result from the predominant use of only an analytical or only an intuitive cognitive process. Because the benefits of judgment aggregation depend strongly on the level of independence among individual judgment errors, aggregating judgments from two different types of cognitive processes—that is, forming crowds with a high level of cognitive-process diversity—should be superior to forming less diverse crowds by aggregating judgments of the same type.^1^ In line with this suggestion, the theoretical results by Herzog and von Helversen (2018) showed that statistically combining the output of exemplar-based and linear-rule-based judgment processes—on which, as we discussed, intuitive and analytical judgments, respectively, might be partially based—provides judgments that are more accurate than those based on only one of the two processes. We thus hypothesized that the predictions of crowds formed by aggregating intuitive and analytical judgments would be more accurate than those of crowds formed by aggregating only analytical judgments (Hypothesis 1a) or than those of crowds formed by aggregating only intuitive judgments (Hypothesis 1b).

Importantly, we also expected that the effect of cognitive-process diversity on crowd accuracy would depend on the size of the crowd. As we outlined previously, the benefits that arise from aggregating judgments depend strongly on the extent to which these judgments bracket the true value. Bracketing, in turn, is more likely to happen when individual judgments are less correlated with each other or when the crowd is larger, but the relationship among these three factors is rather intricate.^2^

In particular, in very small crowds, even if individual judgments were to be completely independent, it is still quite likely that these judgments would frequently not be evenly distributed on both sides of the true value (and thus not bracket the true value) because there are only very few of them. Consequently, the benefits of aggregation in a small crowd will be relatively low, independent of the correlation among individual judgments. By contrast, in large crowds, when judgments are relatively independent, they will be distributed quite evenly around the true value because of the law of large numbers, and bracketing will occur much more frequently. Yet when individual judgments are heavily correlated, they are much more likely to be on the same side of the true value, and so strong bracketing is rather unlikely to happen. Hence, for large crowds we would expect the benefits of aggregation to be very high when judgments are highly independent from each other and very low when judgments are heavily correlated.

On the basis of this line of reasoning, we therefore expected that the positive effects of a lower error correlation (i.e., the effect of cognitive-process diversity) would be stronger for a larger crowd than for a smaller crowd. Overall, this is also strongly consistent with the theoretical results of Lamberson and Page (2012) and Davis-Stober, Budescu, Broomell, and Dana (2015), who formally showed that the relative effect of independence (as measured by the covariance of individual errors) on judgment accuracy increases with crowd size. We thus further hypothesized that judgment accuracy of crowds formed by aggregating analytical and intuitive judgments, relative to the judgment accuracy of crowds formed by aggregating only analytical judgments (Hypothesis 2a) or to the judgment accuracy of crowds formed by aggregating only intuitive judgments (Hypothesis 2b), would be greater in large than in small crowds.

## Study 1

### Method

#### Design and procedure

We recruited 158 participants (90 women, 68 men; mean age = 24 years) at a European university for a laboratory experiment. Six participants did not follow the instructions accurately and were removed from the sample, resulting in a final sample size of 152. Participants were randomly assigned to three conditions: *analytical* (*n* = 48), *intuitive* (*n* = 51), and *control* (*n* = 53).

In all conditions, participants were placed at an individual computer, where they provided answers to 40 questions about the dates of historical events. Each participant received a fixed payment of €6 and could win an additional €6 bonus, calculated on the basis of the judgments’ absolute deviations from the true value. Following prior research (e.g., Dane et al., 2012), we instructed participants in the intuitive condition to base their decisions entirely on their intuition and to avoid consciously thinking about what the right answer was. In addition, participants were given only 7 s to answer each question.^3^ On the other hand, in the analytical condition, we induced participants to make analytical judgments by instructing them to carefully think about the particular reasons for their judgment and to ignore any first impressions or gut instincts that might arise. In addition, participants were given unlimited time to make a judgment. Finally, in the control condition, participants were also given unlimited time, but they were not provided with any specific instructions on how to make their judgments.^4^

#### Measures

##### Crowd accuracy

Crowds were created by randomly selecting (without replacement) individual judges from different conditions and averaging their judgments. We formed four crowd types by drawing judges only from the analytical condition, only from the intuitive condition, only from the control condition, or equally from the analytical and the intuitive conditions. For all crowd types, we also created six crowd sizes: 1, 2, 5, 10, 20, and 48 (the maximum size we could form for all crowd types, determined by our smallest sample size across the three conditions). For example, to form an analytical crowd of 10, we randomly drew 10 judges without replacement from the analytical condition, whereas to form an analytical-intuitive crowd of the same size, five judges were drawn from the analytical and five from the intuitive condition.

For each crowd, we calculated the mean of the individual judgments for each question and determined the corresponding judgment accuracy—defined as the absolute deviation from the true value (e.g., de Oliveira & Nisbett, 2018; Minson, Mueller, & Larrick, 2018; Palley & Soll, 2019).^5^ Specifically, we denoted $x_{ik}^{1},\;x_{ik}^{2},\;.\;.\;.\;x_{ik}^{n}$ as the *n* individual estimates in crowd *k* for question *i*. We computed the corresponding crowd judgment as ${\bar{x}}_{ik}^{n}\;=\;(x_{ik}^{1}\;+\;x_{ik}^{2}\;+\;.\;.\;.\;+\;x_{ik}^{n})\;/\;n$ and accuracy as $\left|a_{ik}^{n}\right|\;=\;\left|{\bar{x}}_{ik}^{n}\;-\;c_{i}\right|$, where *c_i_* is the true value for question *i*. We then averaged the accuracy across all questions as follows: $\left|a_{k}^{n}\right|\;=\;1\;/\;40\;\sum_{i\;=\;1}^{40}\left|a_{ik}^{n}\right|$. We repeated this procedure 10,000 times (i.e., *k* = 1, 2, . . . 10,000) for each crowd type and size, and averaged the results across all trials.

##### Average pairwise correlation

To examine the level of independence between individual judgment errors within a crowd, we measured the average pairwise correlation of signed errors of any two judges who were randomly drawn from either the same or different conditions across multiple trials. Specifically, in a given trial *t*, we randomly picked two judges without replacement—both from the analytical condition, both from the intuitive condition, both from the control condition, or one each from the intuitive and the analytical conditions, respectively. We denoted the estimates of these two judges for question *i* as $x_{it}^{1}$ and $x_{it}^{2}$, where *i* = 1, 2, . . . 40, and *c_i_* represents the correct value. We computed the signed deviations as $a_{it}^{1}\;=\;x_{it}^{1}\;-\;c_{i}$ and $a_{it}^{2}\;=\;x_{it}^{2}\;-\;c_{i}$ and then computed the correlation coefficient of these two sets of signed deviations over all 40 questions. We repeated this procedure 10,000 times and averaged the results.

### Results

Table 1 shows crowd-judgment accuracy aggregated over all questions. We found that across all crowd types, even small crowds of just two provided more accurate judgments than individual judges. Moreover, judgment accuracy increased with crowd size, but once crowds were larger than 20, increasing crowd size further had only a very small effect.

**Table 1.** Absolute Deviations Across Crowd Types and Sizes (Study 1)

Crowd size	Analytical		Intuitive		Control		Analytical-intuitive	
	*M*	95% CI	*M*	95% CI	*M*	95% CI	*M*	95% CI
1^a^	190.12	[152.91, 227.33]	203.44	[166.01, 240.88]	185.72	[158.21, 213.23]	197.05	[160.57, 233.53]
2	149.62	[121.29, 177.95]	159.27	[131.68, 186.86]	144.11	[121.9, 166.32]	141.3	[117.05, 165.55]
5	116.18	[91.69, 140.67]	117.64	[96.56, 138.73]	109.82	[90.32, 129.32]	98.76	[82.16, 115.36]
10	100.61	[76.34, 124.89]	99.16	[78.92, 119.4]	94.23	[74.45, 114.01]	77.07	[62.83, 91.3]
20	92.14	[67.16, 117.12]	88.26	[67.48, 109.04]	84.7	[63.95, 105.46]	63.01	[49.17, 76.86]
48	87.68	[61.8, 113.57]	81.96	[60.05, 103.87]	78.55	[56.42, 100.69]	52.29	[37.63, 66.95]

Note: CI = confidence interval.

a Crowds of size one were created by randomly selecting one individual.

**Fig. 1.**
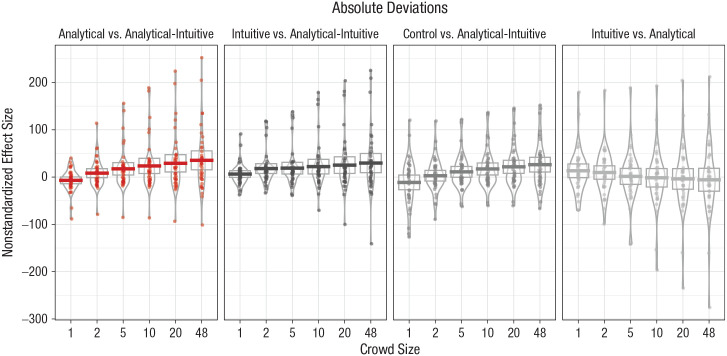
Difference in absolute deviations for each comparison of crowd types, separately for each crowd size (Study 1). Horizontal bars represent mean values, and dots represent individual data points. The height of the boxes denotes 95% confidence intervals, and the width of the irregularly shaped outlines indicates the density of the data.

Rather than relying on null-hypothesis-based tests of our predictions, we followed recommendations by Cumming (2014) and computed effect sizes for the difference in mean absolute deviations between analytical-intuitive crowds and other crowd types and their corresponding 95% confidence intervals (CIs) across different crowd sizes.^6^ Figure 1 presents the results of these comparisons, as well as the comparison between analytical and intuitive crowds.^7^

As shown in Figure 1, we did not find large differences in judgment accuracy between analytical and intuitive crowds of any size. In contrast, providing support for Hypothesis 1, results showed that for all crowd sizes, effect sizes for the comparisons between analytical-intuitive and analytical crowds and between analytical-intuitive and intuitive crowds were in the predicted direction and of similar value. In addition, our results also show that analytical-intuitive crowds were more accurate than control crowds. In line with the predictions of Hypothesis 2 (that the benefits of cognitive-process diversity will be more pronounced in larger crowds), the results in Figure 1 also clearly show that the effect sizes increased in crowd size for the comparison with intuitive crowds (from *M* = 17.97, 95% CI = [7.08, 28.86] years in crowds of two to *M* = 29.65, 95% CI = [8.62, 50.71] years in crowds of 48) and for the comparison with analytical crowds (from *M* = 8.32, 95% CI = [−1.49, 18.12] years in crowds of two to *M* = 35.39, 95% CI = [14.52, 56.27] years in crowds of 48).

Consistent with these findings, our results also showed that the average pairwise correlation ($\bar{r}$) of signed errors was .18, .07, and .16, respectively, for judges drawn from solely the analytical, solely the intuitive, or solely the control condition but only −.03 for judges drawn equally from the intuitive and analytical conditions.

## Study 2

### Method

#### Design and procedure

We recruited 98 participants (51 women, 47 men; mean age = 26 years) from a European university for an online study. Participants were randomly assigned to three conditions (intuitive: *n* = 34, analytical: *n* = 33, and control: *n* = 31) and were asked to estimate the probabilities of the three possible outcomes (Team 1 wins, draw, Team 2 wins) for all 48 matches in the group stage of the 2018 soccer World Cup. Participants were rewarded with course credit and had the opportunity to win an Amazon voucher worth up to €40 depending on their performance, which was assessed after the World Cup on the basis of a quadratic scoring rule. For each match, participants used sliders to enter their probability judgments for all three outcomes; the sliders were programmed so that the stated probabilities always added up to 100%. Our manipulation of participants’ cognitive processes was very similar to that used in Study 1, except that participants in the intuitive condition now had 10 s to enter their estimates for a particular match.

#### Measures

##### Crowd accuracy

As there is no objectively true value for each outcome’s probability, we used probabilities implied in the betting odds provided by sports-betting providers as a benchmark (calculated as the normalized inverse of the provided odds; for details, see the Supplemental Material) and computed the absolute deviation from this benchmark as our measure of judgment accuracy. Betting odds are among the best available predictors for sport events (e.g., Boulier, Stekler, & Amundson, 2006; Spann & Skiera, 2009). They thus constitute an upper bound for judgment accuracy of individuals without very specific expert knowledge, making them an adequate benchmark for the quality of crowd judgments (e.g., Herzog & Hertwig, 2011; Palley & Soll, 2019). We used the same general procedure as in Study 1 to form crowd judgments and compute absolute deviations from the betting-odds benchmark for crowds of different types and of sizes 1, 2, 5, 10, 20, and 31.

##### Brier scores

As a second measure of judgment quality, we also computed Brier scores (Brier, 1950) using the different crowd judgments and the actual final outcome of each game. Specifically, the Brier score for a particular match *i* was calculated as $\sum_{z\;=\;1}^{3}{\left(R_{iz}\;-\;P_{iz}\right)}^{2}$, where $P_{iz}$ de-notes the probability estimate of a particular outcome *z* of match *i*, and $R_{iz}$ is an indicator that equals 1 if the outcome of match *i* is *z* and 0 otherwise.

##### Average pairwise correlation

We followed the same general procedure as in Study 1 to compute signed deviations of two randomly selected judges from probabilities implied in the betting odds. We then calculated the corresponding coefficient of these two sets of signed deviations over all 48 matches for each possible outcome and then averaged across the three outcomes per match. As before, we repeated this procedure 10,000 times and averaged the results.

### Results

**Fig. 2.**
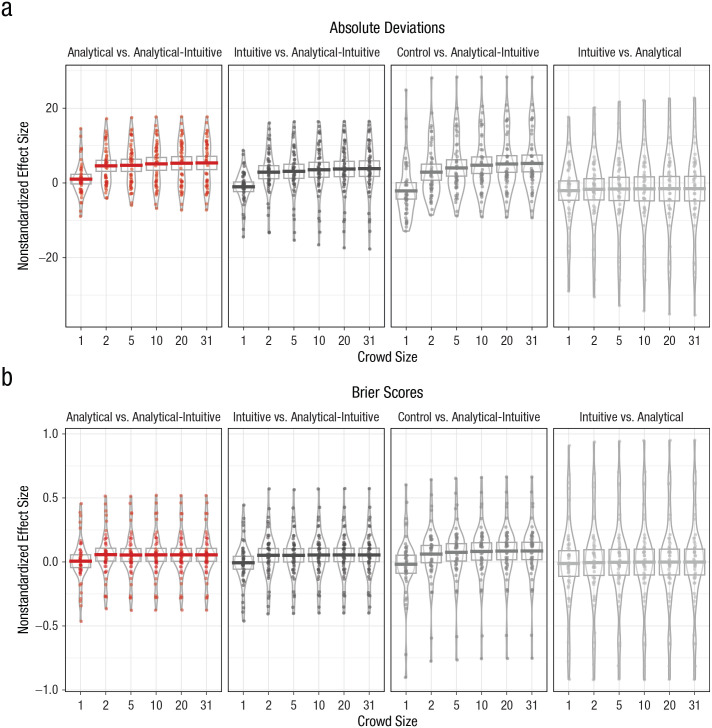
Difference in (a) absolute deviations and (b) Brier scores for each comparison of crowd types, separately for each crowd size (Study 2). Horizontal bars represent mean values, and dots represent individual data points. The height of the boxes denotes 95% confidence intervals, and the width of the irregularly shaped outlines indicates the density of the data.

As shown in Tables 2 and 3, for all crowd types, we found that even small crowds outperformed individual judgments and that absolute deviations and Brier scores decreased with crowd size in a concave fashion. Figure 2 presents the unstandardized effect sizes for the different comparisons of interest with respect to both absolute deviations and Brier scores.

**Table 2.** Absolute Deviations Across Crowd Types and Sizes (Study 2)

Crowd size	Analytical		Intuitive		Control		Analytical-intuitive	
	*M*	95% CI	*M*	95% CI	*M*	95% CI	*M*	95% CI
1^a^	20.95	[18.89, 23.01]	18.90	[17.08, 20.72]	17.80	[14.85, 20.76]	19.93	[18.51, 21.35]
2	18.48	[16.18, 20.78]	16.78	[14.77, 18.80]	16.79	[13.68, 19.89]	13.90	[12.33, 15.47]
5	16.83	[14.31, 19.36]	15.23	[13.02, 17.43]	16.13	[12.92, 19.33]	12.11	[10.32, 13.91]
10	16.24	[13.62, 18.86]	14.66	[12.38, 16.94]	15.88	[12.64, 19.13]	11.13	[9.19, 13.07]
20	15.95	[13.28, 18.62]	14.41	[12.09, 16.73]	15.75	[12.49, 19.02]	10.68	[8.65, 12.70]
31	15.86	[13.17, 18.54]	14.33	[12.00, 16.67]	15.72	[12.44, 18.99]	10.50	[8.44, 12.56]

Note: CI = confidence interval.

a Crowds of size one were created by randomly selecting one individual.

**Table 3.** Brier Scores Across Crowd Types and Sizes (Study 2)

Crowd size	Analytical		Intuitive		Control		Analytical-intuitive	
	*M*	95% CI	*M*	95% CI	*M*	95% CI	*M*	95% CI
1^a^	.83	[.7, .96]	.82	[.69, .94]	.81	[.68, .93]	.82	[.71, .94]
2	.79	[.66, .91]	.78	[.66, .91]	.79	[.67, .91]	.73	[.62, .84]
5	.76	[.63, .89]	.76	[.63, .88]	.78	[.66, .91]	.71	[.60, .81]
10	.75	[.63, .88]	.75	[.63, .88]	.78	[.66, .90]	.70	[.59, .80]
20	.75	[.62, .88]	.75	[.62, .87]	.78	[.66, .90]	.69	[.59, .80]
31	.75	[.62, .88]	.75	[.62, .87]	.78	[.65, .90]	.69	[.59, .80]

Note: CI = confidence interval.

a Crowds of size one were created by randomly selecting one individual.

Figure 2 shows that, as in Study 1, there were only small differences between analytical and intuitive crowds for all crowd sizes. By contrast, supporting Hypothesis 1, results showed that across all crowd sizes, analytical-intuitive crowds exhibited lower absolute deviations than the other three crowd types. Moreover, as predicted in Hypothesis 2, results also showed that effect sizes increased with crowd size, even though this effect was less pronounced than in Study 1. In particular, the mean estimate increased both for the advantage of analytical-intuitive over intuitive crowds in absolute deviations (from *M* = 2.88%, 95% CI = [1.09%, 4.68%] in crowds of two to *M* = 3.83%, 95% CI = [1.69%, 5.97%] in crowds of 31), and for the comparison with analytical crowds (from *M* = 4.58%, 95% CI = [3.07%, 6.10%] to *M* = 5.36%, 95% CI = [3.53%, 7.18%]). Our results for Brier scores showed generally consistent evidence, even though the increase in effect sizes was even less pronounced.^8^

As in Study 1, we also found that the average pairwise correlation of signed errors among judges from the analytical condition ($\bar{r}$ = .79), from the intuitive condition ($\bar{r}$= .83), or from the control condition ($\bar{r}$ = .90) were all higher than the correlation between judges drawn equally from the intuitive and the analytical conditions ($\bar{r}$ = .41).

## Study 3

One potential problem in our previous studies is that each participant made judgments for several items, which might create a dependency between items and thus potentially bias our statistical results.^9^ To address this issue, we aimed in Study 3 to replicate the general findings of Study 1 while asking each participant to make only one judgment, so that judgments across different items would be fully independent from each other. This new design required a very large number of participants who spent only very little time on the study, and we thus opted to conduct our experiment online with participants recruited from Amazon Mechanical Turk. Because running our experiment online gave rise to potential concerns that participants might look up the correct answer on the Internet, we changed our task from the approach used in Study 1 and instead asked participants to estimate the weight of different individuals on the basis of a photograph (e.g., Gino & Moore, 2007). The study was preregistered at OSF (https://osf.io/5k6un/).

### Method

#### Design and procedure

We recruited 3,695 participants with complete responses (1,887 women, 1,808 men; mean age = 37.04 years) from Amazon Mechanical Turk. Participants were compensated with $0.50. In addition to their base compensation, participants could win an additional bonus of $0.25 if their judgment was within 10 pounds of the correct value. All participants had an approval rating of at least 95% and were located in the United States. On average, it took participants 4 min to complete the study. Participants were randomly assigned to three conditions and shown one randomly selected picture of a person (out of 40 different pictures). They were then asked to provide an estimate of this individual’s weight in pounds, measured before breakfast and excluding any clothing. In each condition, we obtained approximately 31 (with a minimum of 30) independent estimates for each of the 40 pictures. Before making a judgment, each participant received general instructions about the task and went through two practice rounds. Participants’ judgment process was manipulated in the same way as in Study 1.

#### Measures

We employed the same procedure as in Study 1 to construct crowd judgments for crowd sizes of 1, 2, 5, 10, 20, and 30 and to assess judgment accuracy.

### Results

**Fig. 3.**
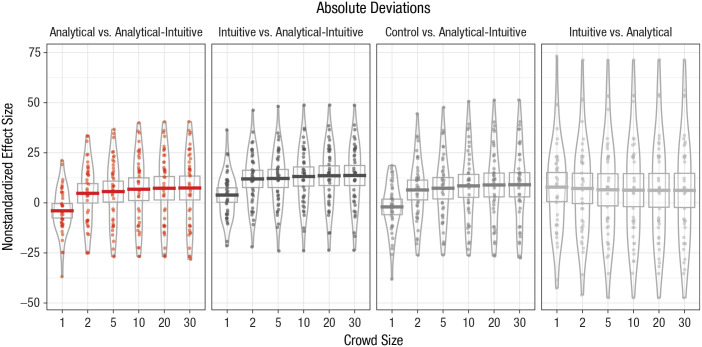
Difference in absolute deviations for each comparison of crowd types, separately for each crowd size (Study 3). Horizontal bars represent mean values, and dots represent individual data points. The height of the boxes denotes 95% confidence intervals, and the width of the irregularly shaped outlines indicates the density of the data.

Table 4 provides an overview of our main results. As in previous studies, we found that even small crowds of any type outperformed individual judgments and that crowd-judgment accuracy increased with crowd size but reached a limit in large crowds. Figure 3 presents nonstandardized effect sizes for different comparisons between crowd types.

**Table 4.** Absolute Deviations Across Crowd Types and Sizes (Study 3)

Crowd size	Analytical		Intuitive		Control		Analytical-intuitive	
	*M*	95% CI	*M*	95% CI	*M*	95% CI	*M*	95% CI
1^a^	31.62	[26.87, 36.37]	39.49	[33.59, 45.39]	33.57	[28.38, 38.75]	35.59	[31.78, 39.39]
2	29.53	[24.47, 34.58]	36.72	[30.74, 42.71]	31.21	[25.71, 36.72]	24.76	[20.18, 29.34]
5	28.09	[22.70, 33.48]	34.63	[28.39, 40.88]	29.75	[23.87, 35.64]	22.45	[17.58, 27.32]
10	27.57	[22.01, 33.13]	33.91	[27.48, 40.34]	29.24	[23.16, 35.31]	20.75	[15.55, 25.96]
20	27.32	[21.66, 32.99]	33.58	[27.01, 40.15]	28.97	[22.79, 35.15]	20.03	[14.66, 25.39]
30	27.21	[21.49, 32.92]	33.45	[26.83, 40.08]	28.84	[22.59, 35.08]	19.78	[14.34, 25.22]

Note: CI = confidence interval.

a Crowds of size one were created by randomly selecting one individual.

The results show that effect sizes for the comparison between analytical-intuitive and analytical, intuitive, or control crowds were all positive, as predicted by Hypothesis 1. Interestingly, we also found that individual judgments in the analytical condition were more accurate, *M* = 7.86 pounds, 95% CI = [0.34, 15.39], than those in the intuitive condition. Consistently, we found that the advantage of analytical-intuitive over intuitive crowds was considerably larger than that over analytical crowds. For example, in large crowds of 30, the relative advantage of analytical-intuitive crowds over intuitive crowds was 13.68 pounds (95% CI = [8.48, 18.87]), compared with only 7.43 pounds (95% CI = [1.23, 13.62]) for the advantage over analytical crowds. Comparing crowds of two and crowds of 30, we again found that mean effect sizes increased for the advantage of analytical-intuitive over intuitive crowds (from *M* = 11.96, 95% CI = [7.46, 16.46] to *M* = 13.68, 95% CI = [8.48, 18.87]) and for that over analytical crowds (from *M* = 4.76, 95% CI = [−0.27, 9.80] to *M* = 7.43, 95% CI = [1.23, 13.62]), as predicted by Hypothesis 2.

## General Discussion

The results of three experimental studies showed that forming crowds with a high level of cognitive-process diversity—by aggregating a combination of intuitive and analytical individual judgments—improved the quality of crowd wisdom, compared with crowds formed by an aggregation of only analytical judgments, only intuitive judgments, or judgments made in a control condition without specific manipulation of judges’ cognitive processes. Moreover, we found that whereas the benefits of cognitive-process diversity generally held for both smaller and larger crowds, the magnitude of these benefits increased with crowd size and eventually approached its maximum as crowds became very large. Providing supporting evidence for the suggestion that the benefits of cognitive-process diversity are driven by higher levels of judgment-error independence, the results of Studies 1 and 2 revealed a lower average correlation in signed errors between judges employing an intuitive cognitive process and those employing an analytical cognitive process, compared with judges relying on the same cognitive process or judges in the control condition.

One particularly interesting finding of Study 3 is that analytical-intuitive crowds still outperformed purely analytical crowds even though individual analytical judgments were more accurate than individual intuitive judgments—implying that in this specific context the benefits of adding more uncorrelated judgments outweighed the detrimental effects of adding less accurate judgments. It is, however, important to note that there are likely a number of domains (e.g., tasks that require the application of formal logic) in which intuitive judgments would be much less accurate than analytical ones and hence adding highly inaccurate though less correlated judgments to a crowd is likely not beneficial (e.g., Mannes et al., 2014).

Previous research has suggested ways to improve judgment aggregation, such as by selecting better performing individuals (e.g., Budescu & Chen, 2014; Mannes et al., 2014) or by refining the aggregation procedure (e.g., Jose & Winkler, 2008; Palley & Soll, 2019). By contrast, our approach focused on increasing independence between individual judgment errors by manipulating the cognitive process employed by individual judges to form their judgments. It thus also complements recent work by de Oliveira and Nisbett (2018), who investigated the possibility of improving crowd wisdom by amplifying the demographic diversity of crowds and found that this approach was largely ineffective. A likely explanation for this difference in results is that we directly manipulated the cognitive process by which judgments were being made, whereas demographic differences frequently might not be associated with differences in individual cognition.

One limitation of our work is that we manipulated judgments to either be predominantly intuitive or predominantly analytical. However, in practice, judgments and decisions might frequently be based on a process in the middle of a continuum with analytical and intuitive processes at the boundaries (e.g., Hammond, 1996). Thus, an interesting direction for future research would be to compare our approach with one in which a crowd is formed by aggregating judgments that are each based on a mixture of analytical and intuitive processes. A related important limitation of our results is that we did not provide direct insights into differences in participants’ exact cognitive processes, such as the use of different judgment rules or reliance on different pieces of information (e.g., Herzog & von Helversen, 2018; Hoffmann et al., 2013). Such differences might explain the higher independence between analytical and intuitive judgments observed in our studies.

A final interesting avenue for future research would be to explore whether our approach toward improving the wisdom of crowds might also help to increase the effectiveness of combining judgments that are made by the same individual (e.g., Herzog & Hertwig, 2009, 2014; Vul & Pashler, 2008).

## Supplemental Material

Keck_OpenPracticesDisclosure_rev – Supplemental material for Enhancing the Wisdom of the Crowd With Cognitive-Process Diversity: The Benefits of Aggregating Intuitive and Analytical Judgments Click here for additional data file. Supplemental material, Keck_OpenPracticesDisclosure_rev for Enhancing the Wisdom of the Crowd With Cognitive-Process Diversity: The Benefits of Aggregating Intuitive and Analytical Judgments by Steffen Keck and Wenjie Tang in Psychological Science

Keck_Supplemental_Material_rev – Supplemental material for Enhancing the Wisdom of the Crowd With Cognitive-Process Diversity: The Benefits of Aggregating Intuitive and Analytical Judgments Click here for additional data file. Supplemental material, Keck_Supplemental_Material_rev for Enhancing the Wisdom of the Crowd With Cognitive-Process Diversity: The Benefits of Aggregating Intuitive and Analytical Judgments by Steffen Keck and Wenjie Tang in Psychological Science

## Transparency

*Action Editor:* Timothy J. Pleskac

*Editor:* D. Stephen Lindsay

*Author Contributions*

Both authors developed the study idea and designed the laboratory experiments. Data were collected and analyzed by S. Keck with input from W. Tang. Both authors jointly wrote the manuscript and approved the final version of the manuscript for submission.

**Open Practices:** The raw data and analysis code from our three studies have been made publicly available via OSF and can be accessed at https://osf.io/847df/. The design and analysis plans for the studies were preregistered at https://osf.io/5k6un/. The complete Open Practices Disclosure for this article can be found at http://journals.sagepub.com/doi/suppl/10.1177/0956797620941840. This article has received the badge for Open Data. The authors preferred not to apply for the Preregistered badge. More information about the Open Practices badges can be found at http://www.psychologicalscience.org/publications/badges.

## Notes

1. This prediction assumes no large differences in judgment accuracy between intuitive and analytical crowds that might outweigh the benefits of aggregating less correlated judgments (e.g., Mannes et al., 2014).

2. In addition, the extent of bracketing and hence the benefits of aggregation might also be affected by each judge’s tendency to systematically under- or overestimate the true values across a number of judgments (Davis-Stober et al., 2015; Davis-Stober, Budescu, Dana, & Broomell, 2014). See the Supplemental Material available online for further information.

3. Although inferring intuitive decisions from short response times has been criticized because many confounding factors can affect response times (Krajbich, Bartling, Hare, & Fehr, 2015), this critique does not apply to the direct manipulation of judgment processes by putting individuals under time pressure (e.g., Evans, Dillon, & Rand, 2015).

4. In all three studies, participants also completed a final survey with demographic questions, a manipulation check with five items assessing participants’ use of different cognitive processes, and measures assessing participants’ self-reported expertise in the task domain (see the Supplemental Material).

5. We also conducted our analysis using a median-based aggregation model and standardized absolute deviations, and we found consistent results (see the Supplemental Material).

6. We also provide results for standardized effect sizes (Cohen’s *d*) in the Supplemental Material.

7. For all three studies, a formal test of our hypotheses with paired-samples *t* tests, Wilcoxon tests, or mixed-effect models can be found in the Supplemental Material available online.

8. Decomposing the Brier score into calibration and resolution showed that the advantage of analytical-intuitive crowds over control crowds originated from a relatively even mix of better resolution and better calibration, and the improvement over analytical and over intuitive crowds was mostly from better resolution only. The detailed results are presented in the Supplemental Material.

9. We provide a detailed analysis of this issue in the Supplemental Material.
