# Supplementary material for: Enhancing the Wisdom of the Crowd With Cognitive-Process Diversity: The Benefits of Aggregating Intuitive and Analytical Judgments
Source: Psychol Sci. 2020 Sep 22;31(10):1272–82. doi: 10.1177/0956797620941840 (PMC7549292; doi:10.1177/0956797620941840)
Supplement: Keck_Supplemental_Material_rev – Supplemental material for Enhancing the Wisdom of the Crowd With Cognitive-Process Diversity: The Benefits of Aggregating Intuitive and Analytical Judgments [file Keck_Supplemental_Material_rev.docx]

**Supplemental Online Materials**

1. **Results for standardized effect sizes**

Table S1

Standardized effect sizes (Cohen’s *d*) across crowd types and sizes

*Note.* Standardized effect sizes are calculated as difference in means divided by the pooled standard deviation.

1. **Direct tests of hypotheses**

Table S2

Results of paired *t*-tests to test hypotheses 1 and 2

Note: Numbers in square brackets denote crowd size;

1. **Brier score decomposition (Study 2)**

Table S3

Results of Brier score decomposition and improvement of mixed crowds across crowd types and sizes in Study 2

*Note*. Measures are averaged across all three outcomes.

1. **Description and analysis of survey items: manipulation checks and participant expertise**

**Items to measure participants’ cognitive process (Study 1, 2, and 3)**

(i) “I based my judgments on my inner feelings and reactions”

(ii) “I made my judgments in a logical and systematic way” (reverse scored)

(iii) “I relied on my gut instinct”

(iv) “I analysed all available information that I could think of in detail” (reverse scored)

(v) “I made judgments mainly based on what felt right to me”

Scale: 1 = “not at all” to 7 = “very much”.

Study 1: $\alpha=.78$; Study 2: $\alpha=.79$; Study 3: $\alpha=.58$.

**Items to measure expertise**

Study 1

(i) “I am very interested in history”

(ii) “I know a lot about different historical events”

(iii) “I spend a lot of time learning about history (e.g., by reading books or watching documentaries)”.

$\alpha=.91$.

Study 2

(i) “I am very interested in professional soccer”

(ii) “I know a lot about different national soccer teams”

(iii) “I spend a lot of time watching international soccer games such as the world cup”.

$$\alpha=.87.$$

Scale: 1 = “not at all” to 7 = “very much”.

**Items to measure perceived difficulty and confidence (Study 3)**

(i) How difficult did you find the task?

Scale: 1 = “extremely easy” to 7 = “extremely difficult”.

(ii) How well do you think you performed on the task compared to other participants?

Scale: best 25%, best 50% but not best 25%, worst 50% but not worst 25%, and worst 25%.

**Results**

**Study 1.** Participants reported to have a medium level of knowledge about historical events **(***M* **=** 3.77, *SD* = 1.40). As expected, we found that participants relied significantly more on an intuitive cognitive process in the intuitive condition (*M* = 5.22, *SD* = 1.12) than in the analytical (*M* = 2.84, *SD* = 0.93), *t*(97) = 11.49, *p* < .001, *d* = 2.31, or control condition, (*M* = 3.25, *SD* = 0.87), *t*(102) = 10.02, *p* < .001, *d* = 1.97. Moreover, participants in the analytical condition also relied less on their intuition than in the control condition, *t*(99) = 2.30, *p* = .024, *d* = 0.46.

**Study 2.** Participants possessed a moderate level of expertise in professional soccer (*M* = 3.78, *SD* = 1.74). As intended participants in the intuitive condition (*M* = 5.14, *SD* = 1.05) relied significantly more on intuitive thinking than in the analytical, (*M* = 2.50, *SD* = 0.74), *t*(65) = 11.88, *p* < .001, *d* = 2.90, or the control condition (*M* = 3.06, *SD* = 1.06), *t*(63) = 7.96, *p* < .001, *d* = 1.98. Moreover, participants relied less on their intuition in the analytical than in the control condition, *t*(62) = 2.44, *p* = . 018, *d* = 0.61.

**Study 3.** Participants perceived the task as moderately difficult (*M* = 3.32, *SD* = 1.67) and the median participant believed to perform in the upper 50% of all participants. Suggesting the success of our manipulation, participants in the intuitive condition (*M* = 4.64, *SD* = 0.90) reported to have relied significantly more on intuitive thinking than in the analytical (*M* = 3.51, *SD* = 0.96), *t*(2486) = 30.21, *p* < .001, *d* = 1.21, or the control condition (*M* = 3.91, *SD* = 0.72), *t*(2487) = 22.23, *p* < .001, *d* = 0.89. Moreover, participants reported having relied less on their intuition in the analytical compared with the control condition, *t*(2411) = 11.62, *p* < .001, *d* = 0.47.

**5. Sample size calculation**

Note that the power of our statistical tests is determined by the number of questions rather than participants in each condition.

**Study 1.**  With an assumed correlation of 0.5 and a medium effect size of 0.5, a power analysis for paired *t*-tests suggested that a total of 40 questions would give us an acceptable statistical power of 0.87. Moreover, assuming a standard deviation of 200 (which we observed in an unrelated prior study with similar knowledge items), a sample size of 50 per condition would allow us to observe the true population mean within an error of ±55 years.

**Study 2.** We limited data collection to two weeks such that all participants relied on similar information. Assuming an effect size of 0.40 observed in Study 1, forty-eight matches provided us with an acceptable power of 0.78. Assuming a standard deviation of 15 percentage points (estimated based on a small pilot study with 9 participants), we had 30–35 observations in each condition to estimate the true population mean for each item within 5 percentage point.

**Study 3.**  Based on the values observed in Study 1, a paired *t*-tests with 40 questions would provide a satisfactory statistical power of 0.92. Under the assumption that responses are normally distributed with a standard deviation of 29 (which we observed in a pilot study) obtaining 30 observations for each question allows us to estimate the true population mean for each question within an interval of +/- 11 pounds.

**6. Descriptive overview of individual judgments across questions and conditions**

Table S4: Descriptive overview of results in Study 1

Table S5

Descriptive overview of results in Study 2

Table S6

Descriptive overview of results in Study 3

|  |  |  |  |  |  |  |  |  |  |  |  |
| --- | --- | --- | --- | --- | --- | --- | --- | --- | --- | --- | --- |
|  |  | Correct value |  | ANL | |  | INT | |  | CON | |
| Picture |  |  |  | *M* | *SD* |  | *M* | *SD* |  | *M* | *SD* |
| 1 |  | 95 |  | 131.93 | 18.14 |  | 78.33 | 21.28 |  | 122.13 | 17.72 |
| 2 |  | 97 |  | 132.87 | 14.20 |  | 120.56 | 10.60 |  | 137.94 | 23.15 |
| 3 |  | 106 |  | 132.50 | 21.19 |  | 74.13 | 45.91 |  | 111.20 | 12.53 |
| 4 |  | 106 |  | 195.03 | 26.35 |  | 147.65 | 29.58 |  | 182.93 | 20.96 |
| 5 |  | 117 |  | 170.73 | 28.16 |  | 98.55 | 19.26 |  | 171.39 | 22.66 |
| 6 |  | 117 |  | 93.32 | 10.57 |  | 120.23 | 51.55 |  | 92.71 | 19.05 |
| 7 |  | 119 |  | 105.97 | 14.00 |  | 203.38 | 40.62 |  | 128.07 | 19.81 |
| 8 |  | 121 |  | 140.57 | 20.09 |  | 96.80 | 13.05 |  | 162.48 | 27.36 |
| 9 |  | 123 |  | 160.40 | 14.68 |  | 130.50 | 24.04 |  | 157.63 | 23.82 |
| 10 |  | 132 |  | 174.50 | 19.14 |  | 165.45 | 20.22 |  | 178.97 | 26.13 |
| 11 |  | 135 |  | 121.67 | 31.38 |  | 139.88 | 12.45 |  | 116.03 | 13.13 |
| 12 |  | 137 |  | 110.70 | 15.78 |  | 183.73 | 22.14 |  | 102.63 | 19.61 |
| 13 |  | 139 |  | 167.27 | 24.65 |  | 103.19 | 16.39 |  | 164.43 | 32.11 |
| 14 |  | 139 |  | 129.07 | 17.18 |  | 106.70 | 20.17 |  | 129.93 | 25.65 |
| 15 |  | 146 |  | 129.30 | 25.23 |  | 159.87 | 25.73 |  | 138.90 | 19.92 |
| 16 |  | 146 |  | 116.27 | 18.32 |  | 93.13 | 19.08 |  | 119.55 | 24.28 |
| 17 |  | 148 |  | 161.50 | 15.30 |  | 167.68 | 24.85 |  | 146.97 | 27.89 |
| 18 |  | 150 |  | 128.73 | 17.93 |  | 177.81 | 42.68 |  | 132.83 | 21.43 |
| 19 |  | 154 |  | 178.03 | 21.94 |  | 118.94 | 37.09 |  | 183.90 | 19.74 |
| 20 |  | 157 |  | 156.23 | 23.64 |  | 186.50 | 35.87 |  | 166.90 | 17.27 |
| 21 |  | 157 |  | 189.87 | 26.89 |  | 111.88 | 24.36 |  | 193.20 | 25.88 |
| 22 |  | 159 |  | 204.33 | 21.96 |  | 134.00 | 22.57 |  | 199.73 | 19.74 |
| 23 |  | 165 |  | 156.13 | 19.30 |  | 126.61 | 29.72 |  | 153.32 | 32.63 |
| 24 |  | 172 |  | 173.30 | 32.76 |  | 229.58 | 31.58 |  | 173.87 | 28.82 |
| 25 |  | 174 |  | 152.63 | 19.87 |  | 218.59 | 18.80 |  | 147.47 | 18.86 |
| 26 |  | 176 |  | 141.47 | 21.96 |  | 201.44 | 43.02 |  | 141.20 | 20.92 |
| 27 |  | 179 |  | 176.30 | 16.84 |  | 145.97 | 29.06 |  | 178.29 | 24.37 |
| 28 |  | 181 |  | 210.00 | 21.66 |  | 169.23 | 25.98 |  | 199.61 | 16.56 |
| 29 |  | 183 |  | 142.57 | 24.37 |  | 213.00 | 50.83 |  | 140.03 | 26.09 |
| 30 |  | 183 |  | 192.77 | 33.13 |  | 184.61 | 16.45 |  | 189.94 | 23.59 |
| 31 |  | 183 |  | 165.60 | 27.61 |  | 111.80 | 21.61 |  | 159.00 | 22.04 |
| 32 |  | 187 |  | 152.45 | 22.05 |  | 185.81 | 24.21 |  | 154.13 | 21.15 |
| 33 |  | 187 |  | 171.77 | 20.36 |  | 225.73 | 22.84 |  | 177.97 | 21.92 |
| 34 |  | 190 |  | 180.93 | 37.91 |  | 232.74 | 47.51 |  | 171.43 | 31.63 |
| 35 |  | 194 |  | 152.90 | 22.02 |  | 206.91 | 51.83 |  | 139.48 | 24.08 |
| 36 |  | 201 |  | 148.77 | 26.24 |  | 229.91 | 33.64 |  | 137.93 | 18.17 |
| 37 |  | 209 |  | 148.40 | 31.81 |  | 140.77 | 44.37 |  | 140.42 | 22.09 |
| 38 |  | 214 |  | 174.03 | 24.08 |  | 142.03 | 22.92 |  | 150.55 | 26.17 |
| 39 |  | 229 |  | 219.23 | 20.36 |  | 196.19 | 26.66 |  | 198.69 | 34.44 |
| 40 |  | 232 |  | 211.80 | 26.50 |  | 158.55 | 25.50 |  | 204.73 | 23.87 |
|  |  |  |  |  |  |  |  |  |  |  |  |

**7. Robustness checks**

Table S7

Effect sizes (non-standardized) for median-based aggregation

Note: Crowd judgments were formed using the same general procedure as described in the main manuscript, but were computed using the median judgment in a particular crowd rather than the mean.

Table S8

Effect sizes (non-standardized) from mean-based aggregation with standardized absolute deviations

Note: Crowd judgments were formed using the same general (mean-based procedure) as described in the main manuscript. However, effect sizes are based on standardized absolute deviations.

**8. Alternative tests of hypotheses**

Table S9

Results of Wilcoxon tests of Hypotheses 1 and 2

Table S10

Results of mixed models to Hypotheses 1 and 2

**9. Development of experimental stimuli**

**Study 1.** To compile the list of questions used in Study 1, we searched for “famous events in history” on Google. The final list of events was then compiled from several websites meeting the following two criteria: (i) all events taking place after the year 1000 and before the year 1900, and (ii) at least two events taking place in each century (except for the 12^th^ century where two events taking place in 1088 and 1206 were selected instead). Overall, the websites listed considerably more events in later centuries, and hence the list included more events happening later in history.

**Study 2.** We chose the complete list all 48 matches in the group stage of the 2018 soccer World Cup. Choosing games in the group stage rather than the knock-out stage had the advantage that the different fixtures were known several months in advance.

**Study 3.** We collected the full-body frontal pictures of 40 adults (24 men and 16 women), with heads of the individuals blurred by us to preserve their anonymity. In addition, we also used JavaScript to disable the browser option of downloading or automatically searching for the picture on the internet. The pictures showed personal acquaintances of the authors and of a research assistant who helped us with creating the study materials. The pictures were either directly provided to us by these acquaintances or in some cases taken by the authors or the research assistant. All persons whose pictures were included in the study were aware of the general procedure of the study and gave their permission to use their picture in the study.

**10. Results on individual signed bias**

To analyze to what extent our manipulation might have affect individuals’ tendency to systematically over or underestimate the true value across questions, we computed individuals` signed deviation from the true value for each question in the different conditions.

Our results show that on average the individual signed deviation is positive for analytical judgments ($M=18.89, SD=110.73$), close to zero for judgments in the control condition ($M=-4.07, SD=95.24$) and negative for intuitive judgments ($M=-54.05, SD=101.17$). For Study 2 this type of analysis is not applicable: in Study 2 an individual’s probability judgments across the three outcomes always had to sum to 100%, and hence it is not possible for participants to exhibit a consistent bias in one direction. In Study 3 each individual only made one judgment and thus we could not measure individual-level biases across questions. However, we can still compute to what extent the mean values across all participants are below or above the true value in the different conditions. Our analysis shows that signed deviations from the true value averaged across all questions are quite close to zero for judgments in the analytical (*M* = -0.93, *SD* = 32.83) the intuitive (*M* = -2.51, *SD* = 39.64) as well as the control (*M* = -3.51, *SD* = 34.94) condition.

To provide further insights into the extent to which our manipulation might have caused participants to over- or underestimate the true values, we also conducted a simple analysis in which out of all questions we first counted the number of items where the mean of the intuitive condition and that of the analytical condition are on different sides of the true value, i.e., the two means bracket the true value. Within this subset of questions, we then counted the number of items for which the mean of intuitive judgments was below and that of analytical judgments was above the true value *c* (i.e., $M_{ANL}>c \text{and} M_{INT}<c$), as well as the number of items for which we observed the opposite pattern (i.e., $M_{ANL}<c \text{and} M_{INT}>c$). The results across all three studies are shown in Table S11 below.

Table S11

Bracketing patterns based on mean values in analytical or intuitive condition

|  |  |  |  |  |  |  |
| --- | --- | --- | --- | --- | --- | --- |
|  |  | Total number of items |  | Number of items *M*_ANL_ > *c* and *M*_INT_ < *c* |  | Number of items *M*_ANL_ < *c* and *M*_INT_ > *c* |
| Study 1 |  | 40 |  | 14 |  | 9 |
| Study 3 |  | 40 |  | 9 |  | 14 |
|  |  |  |  |  |  |  |

These results provide some evidence that parts (but not all) of the advantage of ANL-INT crowds might arise from differences in the overall level of bias between intuitive and analytical judgments. In particular intuitive judgments were somewhat more likely to overestimate the true value than analytical judgments in Study 1, and somewhat more likely to underestimate the true value in Study 3.

**11. Calculation of benchmark probabilities in Study 2**

During the same two weeks before the start of World Cup, we obtained the decimal betting odds for the three possible outcomes of each game from the websites of two major online sports betting firms, “Pinnacle” and “Betways”. We then averaged the odds provided on the two respective websites and computed the probabilities (rounded to the next integer value) for each outcome implied in these odds. In order for sport betting firms to be profitable, their provided odds should be equal to the inverse of the probability of a particular outcome plus an additional profit margin called “overround” which for our two data sources is typically around 3–5%. Thus, to obtain the implied probabilities we first calculated the inverse of the decimal odds, and then normalized the three implied probabilities by dividing each probability by the sum of the three probabilities.

**12. Effect of individual-level bias on Type I error in Study 1**

Imagine that we have $N$ questions and $M$ individuals, where each individual answers all $N$ questions. The error matrix with element $\eta_{i}^{j}$, $i\in\{1,2,\ldots, M\}$, $j\in\{1,2,\ldots, N\}$, is expressed as follows:

$$M \text{individuals}$$

$$N \text{questions}\left( \begin{matrix} \eta_{1}^{1} & \cdots& \eta_{M}^{1} \\ \vdots& \ddots& \vdots\\ \eta_{1}^{N} & \cdots& \eta_{M}^{N} \end{matrix} \right)$$

We further assume that each individual’s error is the sum of individual bias and random error, i.e.,

$\eta_{i}^{j}=x_{i}+\epsilon_{i}^{j},$ (1)

or alternatively in matrix format,

$$\left( \begin{matrix} \eta_{1}^{1} & \cdots& \eta_{M}^{1} \\ \vdots& \ddots& \vdots\\ \eta_{1}^{N} & \cdots& \eta_{M}^{N} \end{matrix} \right)=\left( \begin{matrix} x_{1} & \cdots& x_{M} \\ \vdots& \ddots& \vdots\\ x_{1} & \cdots& x_{M} \end{matrix} \right)+\left( \begin{matrix} \epsilon_{1}^{1} & \cdots& \epsilon_{M}^{1} \\ \vdots& \ddots& \vdots\\ \epsilon_{1}^{N} & \cdots& \epsilon_{M}^{N} \end{matrix} \right).$$

Finally, we assume that both $x_{i}$ and $\epsilon_{i}^{j}$ are independent normally distributed variables, where

$$x_{i}\sim N\left( 0,s_{1}^{2} \right), \text{and} \epsilon_{i}^{j}\sim N\left( 0,s_{2}^{2} \right).$$

Based on this model we tried to assess how frequently we would find confirming evidence (at the p < .05 level) for our hypothesis that ANL-INT crowds are more accurate than INT or ANL crowds purely due to chance when there is actually no difference between judgments in the INT and ANL conditions.

We ran our simulation with 100 simulated participants and 40 questions (the number of questions we used in Study 1), i.e., $M=100$ and $N=40$, and systematically varied the values of $s_{1}^{2}$ and $s_{2}^{2}$. Based on the described model, we first simulated errors for each individual and each question. We randomly sampled 50 columns from the error matrix to simulate participants’ errors in the analytical condition, and let the rest of the 50 columns be participants’ errors in the simulated intuitive condition. To compose an analytical (resp., intuitive) crowd of size $k$, we randomly sampled $k$ participants from the simulated analytical condition (resp., simulated intuitive condition) and averaged their errors for each question. Moreover, to compose an ANL-INT crowd of size $k$, we randomly sampled $k/2$ participants from the simulated analytical condition and $k/2$ participants from the simulated intuitive condition, and averaged their errors for each question.

For each of the three crowd types we then conducted paired *t*-tests to compare the accuracy (absolute value of the signed error) between ANL-INT and ANL crowds, and between ANL-INT and INT crowds. We repeated this procedure 10000 times and counted the number of times that we observed a significant advantage ($p < .05$) of ANL-INT crowds over ANL or INT crowds as we predict in Hypotheses 1a and 1b respectively.

Our initial simulations suggested that the results differ only very slightly across crowd sizes, and depend on the ratio of $s_{1}$ and $s_{2}$ rather than their absolute values. Hence, below we show the results for a crowd size of 10 and different ratios of $s_{1}/s_{2}$.

Table S12

Proportion of times that ANL-INT crowds outperform one (ANL or INT) or both crowds (ANL and INT) crowds, corresponding effect sizes, and average pairwise correlation between questions based on simulated errors; $k=10$.

As the results in Table 12 show, the proportion of false rejections of H0 (Type 1 errors) very strongly depends on the ratio of $s_{1}$ and $s_{2}$. When $s_{1}/s_{2}=0$, i.e., when individuals do not have a constant systematic bias in their estimates, the observed proportion of false rejections of the null hypothesis for the independent comparisons with ANL or INT crowds is consistent with the theoretically expected value of 0.025. As the ratio $s_{1}/s_{2}$ increases, the observed proportion of false rejections of H0 also increases. We thus find that Type 1 Errors can indeed be inflated due to the presence of a systematic individual bias. However, it is also important to note that in this case the average effect size across all simulations is always close to zero, which means that although the rate of Type 1 errors is higher than it should normatively be, this issue does not cause a directional bias in favour of ANL-INT crowds.

Importantly, in the last column of Table 12 we also report the average pairwise correlation between the 40 questions that are implied by the different s1/s2 ratios which unlike the exact values of s1 and s2 we could directly measure in our actual sample from the laboratory experiment in Study 1. In particular, across all three conditions the average pairwise correlation between the 40 questions was 0.087. As shown in Table 12 this value would approximately correspond to an $s_{1}/s_{2}$ ratio of between 0.25 and 0.33. Thus, based on the simulation model this would imply that in Study 1 the probability of the null hypothesis to be falsely rejected for one of the two comparisons would be between approximately 0.031 and 0.042 and the probability that we falsely conclude that ANT-INT crowds significantly outperform both ANT and INT crowds would be between 0.006 and 0.008.

Finally, based on the observed average pairwise correlation of 0.087 from our laboratory sample, we also conducted a more direct simulation in which in each iteration we simulated 40 correlated random variables following a multivariate normal distribution with mean zero and constant correlation of 0.087, representing the 40 correlated errors of the questions; each random variable has 100 draws, representing errors from 100 participants. Like before, we randomly split the 100 observations in two parts to simulate the intuitive and analytical conditions, formed ANL, INT, and ANL-INT crowds, and computed the corresponding absolute value of crowd errors. Consistent with our previous results, results only marginally differ across crowd sizes. Moreover, in line the previous findings across all crowd sizes we find an average false positive rate of 0.037 for the independent comparisons with ANL-INT crowds with ANL or INT crowds and an average false positive rate of 0.007 for the hypothesis that ANL-INT significantly outperform both ANL and INT crowds.

In summary, our analysis suggests that although dependence in our observations might indeed be present and to affect the observed *p*-values, this effect is likely only relatively small and our main findings are very unlikely to be purely driven by this dependence.

We are not able to repeat the same procedure for Study 2, because the stated probabilities across the three outcomes always had to sum up to 100%, and thus an individual could not systematically over- or underestimate our benchmark value (the probabilities implied in the betting odds) across all three possible outcomes. For example, if an individual systematically *overestimates* the probability of winning for one team, then at the same time he or she systematically *underestimates* the probability that the other team wins (and/or the probability of a draw). Hence, it is not clear how to assign a bias to an individual in this case. Similarly, it is not meaningful to compute the average pairwise correlation across items in this case.
